# Thank you to the reviewers of *Rheumatology Advances in Practice* 2023

**DOI:** 10.1093/rap/rkae006

**Published:** 2024-01-30

**Authors:** Ai Lyn Tan

**Affiliations:** NIHR Leeds Biomedical Research Centre, Leeds Teaching Hospitals NHS Trust, Leeds, UK; Leeds Institute of Rheumatic and Musculoskeletal Medicine, University of Leeds, Leeds, UK

2023 has been a much anticipated milestone for *Rheumatology Advances in Practice* (RAP) – achieving the journal’s first impact factor, coming in at an impressive 3.1. This is cause for celebration and is a culmination of the dedication and hard work of the team behind the success of RAP and the wider RAP family, which includes not only our editorial team, associate editors and Editorial Board members, but also our authors and reviewers.

The RAP reviewers have been key to the success of the journal; their selfless commitment in helping to ensure the quality of our publications is very much appreciated. Our reviewers enjoy a fulfilling satisfaction of realizing their valuable input contributed to our inaugural impact factor. Together with the authors and reviewers, we are excited about the future of the journal and the benefits our publications will bring to the rheumatology community and patients.

This year, our reviewers also supported a number of RAP initiatives. Our 2^nd^ Trainee Publishing Programme proved to be popular again, with the five winning entries receiving free open access publication [1]. We produced our first special issue on ‘Exercise as medicine’ [2], led by Prof. Lindsay Bearne, one of our fantastic associate editors. We also published our new series, ‘Current and future advances in practice’, led by Dr David Liew, who is a member of the RAP Editorial Board and Digital and Social Media Committee [3]. These initiatives define a number of principles RAP believes in. First, being inclusive. Last year RAP introduced lay summaries to our publications by considering readability for our patients and non-specialist readers [4]. The Trainee Publishing Programme recognizes the importance of providing future generations of experts the platform to publish and provides these emerging researchers with an encouraging opportunity to do so. The new ‘Current and future advances in practice’ series offers a platform for less well-studied rheumatology conditions to be highlighted, as these apparently underserved diseases are especially important to the patients who live with them. Second, RAP encourages novel ideas and initiatives from its Editorial Board members and provides opportunities for members to extend themselves in leading these projects.

As RAP continues to be creative in being inclusive and in providing opportunities to do so, this ambition relies on our reviewers and their insights, expertise and recommendations. We would therefore like to thank all our reviewers for their unwavering support and hope to continue to welcome more reviewers into the RAP family.

## Data Availability

No new data were generated or analysed in support of this article.

## List of reviewers

Abhishek Abhishek

AC Dens

Ade Adebajo

Ai Lyn Tan

Aimie Patience

Alain Lescoat

Albrecht Bertrains

Alejandro Balsa

Alessandro Giollo

Ali Jawad

Alvise Berti

Anastasia-Vasiliki Madenidou

Andrea Di Matteo

Antoni Chan

Arianna D’Antonio

Arshid Bhat

Arvind Kaul

Balaxs Odler

Bart Spaetgens

Bente Glintborg

Bernadette Brady

Bhaskar Dasgupta

Catherine Hill

Charlotte Sharp

Chee-Seng Yee

Chen Li

Chih-Wei Tseng

Christoph Baerwald

Christoph Fiehn

Christopher Edwards

Christopher Joyce

Clio Ribbens

Corinne Fisher

Coziana Ciurtin

Cristiana Sieiro Santos

Dae-Hyun Yoo

Dale Fournier

Dan Kelleher

Daniël Blockmans

Daniela Di Giuseppe

David Liew

Deepak Nagra

Deniz Bayraktar

Diederik De Cock

Durga Misra

Eleonoora Hintsa Figueiredo

Elise van Mulligen

Ellen De Langhe

Elliott Lever

Emre Bilgin

Enrico De Lorenzis

Eugene Lipov

Evelien Gielen

Fateme Zare

Fiona Pearce

Francesco Bertoldo

Francisco Ortiz-Sanjuán

Gabriel Horta-Baas

George Bertsias

George Fragoulis

Gizem Ayan

Gwenda Simons

Haner Direskeneli

Hanna Johnsson

Heather Rostron

Helen Keen

Helen Parsons

Helena Enocsson

Helena Marzo-Ortega

Hema Chaplin

Hennie Raterman

Hiroyuki Wakiguchi

Ho So

Hubert de Boysson

Ilfita Sahbudin

Ilke Coskun Benlidayi

Ingris Peláez-Ballestas

Isabela Borges

Jacopo Ciaffi

Jade Skeates

James Galloway

Janice Mooney

Jean-Baptiste Vulsteke

Jon Barratt

Julie Dawson

Julien Haroche

Karen Walker-Bone

Karim Raza

Karolina Gente

Katie Druce

Katie Hackett

Katie Mageean

Katrien Devusser

Kavita Makan

Keegan Knittle

Keith Lim

Kristen Young

Kulveer Mankia

Kunal Chandwar

Latika Gupta

Laura Andreoli

Laura C. Coates

Laura Ross

Laure Gossec

Leslie Schrieber

Lindsey Cherry

Linn Karlsson

Louise Warburton

Luca Quartuccio

Manouk de Houge

Marc Scherlinger

Marco Di Battista

Marco Krasselt

Marco Matucci-Cerinic

María Carmen Garcia-Gomez

Marie Falahee

Marie Vermant

Mark Garton

Mark Russell

Mathurin Fumery

Mats Junek

Matthew Baker

Max Yates

Md Yuzaiful Md Yusof

Michael Backhouse

Michaël Doumen

Miguel Angel González-Gay

Mohit Goyal

Motohisa Yamamoto

Muhammad Nisar

Murat Torgutalp

Mwidimi Ndosi

Natalie McCormick

Natasha Cox

Neelam Pery

Neil Basu

Nicola Mondanelli

Norelee Kennedy

Owen Cronin

Ozgur Kasapcopur

Pauline Ho

Peter Korsten

Petra De Haes

Pierre Philippet

Rabia Miray Kisla Ekinci

Raouf Hajji

Rebecca Nicolai

Rene Westhovens

Ricardo Ferreira

Richard Conway

Richard Watts

Rigetu Zhao

Rik Lories

Roba Ghossan

Robert Biesen

Roberta Ramonda

Rosalinde Stal

Ruth Wittoek

Saion Chatterjee

Samantha Hider

Samir Patel

Sander van Leuven

Sandra Robinson

Sarah Dyball

Sarah Mackie

Sebastian Sattui

Sham Santhanam

Sheilla Achieng

Shih-Yang Chen

Shramana Ghosh

Silvia Piantoni

Simone Parisi

Sizheng Steven Zhao

Sofia Pazmino

Stephanie Shoop-Worrall

Takashi Kida

Tânia Santiago

Tao Yue

Tate Johnson

Tim L. Jansen

Titilola Falasinnu

Toby Helliwell

Torsten Diekhoff

Valentin Schäfer

Victoria Easton

Victoria Flower

Victoria Menzies

Vinod Ravindran

Vivekanand Tiwari

Wanlong Wu

Wei Jiang

Wenjia Sun

William Gregory

William Tillett

Xavier Puechal

Xiaoming Shu

Xin-Yue Xiao

Xinping Tian

Yeboah Kwaku Opoku

Yeliz Prior

Yongpeng Ge

Yumin Zheng

Yves Piette

Zsuzsanna McMahan
